# Palbociclib and Fulvestrant Act in Synergy to Modulate Central Carbon Metabolism in Breast Cancer Cells

**DOI:** 10.3390/metabo9010007

**Published:** 2019-01-02

**Authors:** Benedikt Warth, Amelia Palermo, Nicholas J.W. Rattray, Nathan V. Lee, Zhou Zhu, Linh T. Hoang, Yuping Cai, Anthony Mazurek, Stephen Dann, Todd VanArsdale, Valeria R. Fantin, David Shields, Gary Siuzdak, Caroline H. Johnson

**Affiliations:** 1 The Scripps Research Institute, Scripps Center for Metabolomics and Mass Spectrometry, 10550 North Torrey Pines Road, La Jolla, CA 92037, USA; benedikt.warth@univie.ac.at (B.W.); palermoa@scripps.edu (A.P.), lhoang@scripps.edu (L.T.H.), siuzdak@scripps.edu (G.S.); 2 Department of Food Chemistry and Toxicology, Faculty of Chemistry, University of Vienna, Währingerstraße 38, 1090 Vienna, Austria; 3 Vienna Metabolomics Center (VIME), University of Vienna, 1090 Vienna, Austria; 4 Department of Environmental Health Sciences, Yale School of Public Health, Yale University, New Haven, CT 06511, USA; nicholas.rattray@yale.edu (N.J.W.R.), ping.cai@yale.edu (Y.C.); 5 Oncology Research, Pfizer Worldwide Research and Development, San Diego, CA 92121, USA; Nathan.V.Lee@pfizer.com (N.V.L.); Zhou.Zhu@pfizer.com (Z.Z.); Anthony.Mazurek@pfizer.com (A.M.); Stephen.Dann@pfizer.com (S.D.); Todd.VanArsdale@pfizer.com (T.V.); valeria.fantin@oricpharma.com (V.R.F.); David.Shields@pfizer.com (D.S.)

**Keywords:** combination drug therapy, breast cancer, multi-omics, metabolomics, RNA-seq, XCMS Online

## Abstract

The aims of this study were to determine whether combination chemotherapeutics exhibit a synergistic effect on breast cancer cell metabolism. Palbociclib, is a selective inhibitor of cyclin-dependent kinases 4 and 6, and when patients are treated in combination with fulvestrant, an estrogen receptor antagonist, they have improved progression-free survival. The mechanisms for this survival advantage are not known. Therefore, we analyzed metabolic and transcriptomic changes in MCF-7 cells following single and combination chemotherapy to determine whether selective metabolic pathways are targeted during these different modes of treatment. Individually, the drugs caused metabolic disruption to the same metabolic pathways, however fulvestrant additionally attenuated the pentose phosphate pathway and the production of important coenzymes. A comprehensive effect was observed when the drugs were applied together, confirming the combinatory therapy’s synergism in the cell model. This study also highlights the power of merging high-dimensional datasets to unravel mechanisms involved in cancer metabolism and therapy.

## 1. Introduction

Cell cycle regulation is frequently disrupted in breast cancer [1,2]. Cyclin-dependent kinases (CDKs) control this regulation enabling quiescent cells to enter the G_1_-phase and transition to the S phase. CDKs 4 and 6 phosphorylate the retinoblastoma (RB) protein enabling the release of E2F transcription factors (E2Fs) which mediates transition into the S-phase. Mutations to the CDK-RB1-E2F pathway typically result in the amplification of *CCND1* which encodes cyclin D1. Both are correlated with estrogen receptor positive (ER+) breast cancers. Thus, the re-establishment of normal cell cycle control through the inhibition of CDKs is an interesting option for the development of targeted cancer therapy. In recent years, agents have been developed which selectively target CDK4/6 [2].

The development of CDK4/6 inhibitors such as palbociclib (Ibrance^®^, PD0332991) target the adenosine triphosphate (ATP) binding site of CDK4-cyclin D and CDK6-cyclin D complexes. This induces cell cycle arrest in the G_1_-phase [3]. Palbociclib is a selective, small-molecule inhibitor of CDK4/6 with the ability to block RB phosphorylation. It can be taken in combination with either the aromatase inhibitor letrozole, or the ER antagonist fulvestrant. Letrozole is used as an initial endocrine-based therapy in post-menopausal women with ER+, human epidermal growth factor receptor 2 (HER2) negative metastatic breast cancer [4,5]. An accelerated U.S. food and drug administration (FDA) approval for this combined therapy was granted in February 2015. This was based on a randomized phase 2 study of 165 post-menopausal women, which showed a progression-free survival (PFS) rate of about 20.2 months when patients were treated with palbociclib and letrozole, compared to a PFS rate of 10.2 months among those treated with letrozole alone [6]. In early 2016, the FDA approval for palbociclib was expanded to include combined therapy with fulvestrant, based on a phase 3 study [7]. The median PFS of 3.8 months (placebo/fulvestrant) was increased to 9.2 months for palbociclib/fulvestrant [7]. This combined treatment is currently in use for women with disease progression following hormonal therapy and can also be applied to pre-menopausal women [8].

In vitro studies have demonstrated the sensitivity of palbociclib towards different breast cancer cell lines and showed a synergistic effect with tamoxifen and trastuzumab in ER+ and HER2-amplified cell lines, respectively [9]. In addition, a previous study investigated the effect of calcein acetoxymethyl-ester (a potent inhibitor of CDK4/6) on cancer cell metabolism, revealing an associated decrease in the concentration of sugar phosphates. This results in an imbalance of the pentose phosphate pathway (PPP) towards the non-oxidative branch versus the oxidative in human colon adenocarcinoma cells, and a state of metabolic inefficiency hypothesized to halt cell proliferation [10]. We also recently employed metabolomics to investigate the effect of palbociclib and letrozole used in single and combination doses in MCF-7 cells. We determined that the combined effects of palbociclib and letrozole on cellular metabolism had a more profound effect than each agent alone, with enhanced changes seen in metabolites in nucleotide metabolism, amino acids, and central carbon metabolism [11].

Therefore, the aims of our study were to determine whether the combined effects of palbociclib and fulvestrant also exert a synergistic effect on breast cancer cell metabolism, and help understand the mechanism underlying the increase in PFS for patients undergoing combined CDK4/6 inhibitor and endocrine therapies. We integrated metabolomic and transcriptomic data using XCMS Online to provide a multi-omic view of dysregulated metabolic pathways [12]. This revealed the response of both metabolites and genes to each drug, and the combined effect of attenuating multiple pathways arresting cell growth.

## 2. Results and Discussion

### 2.1. Metabolomics Analysis

**Figure 1:**
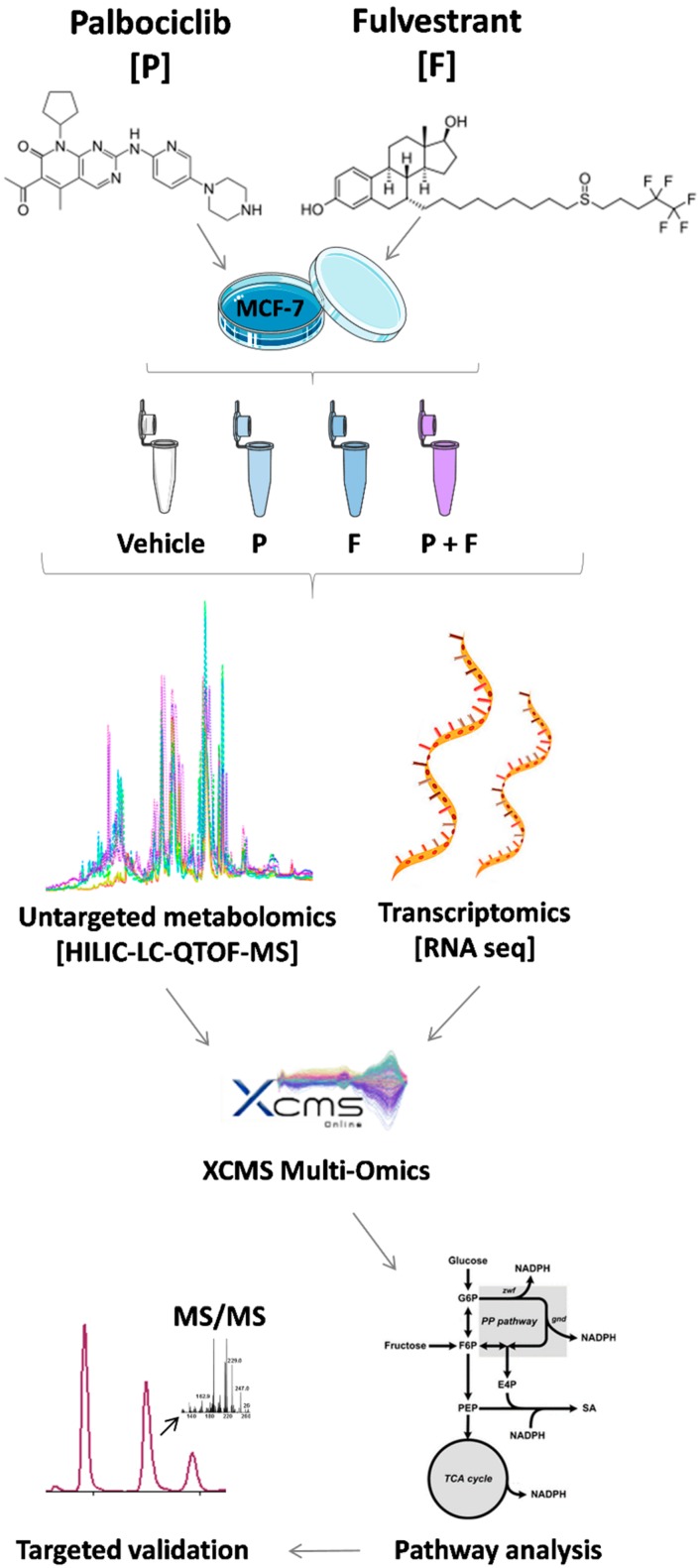
Overview of the workflow applied. MCF-7 breast cancer cells were treated either with the Cyclin-dependent kinases (CDK)4/6 inhibitor palbociclib, the estrogen receptor antagonist fulvestrant, or a combination of both drugs. Cells were extracted and analyzed after two days, seven days (without refeed), and seven days (with refeed).

**Figure 2:**
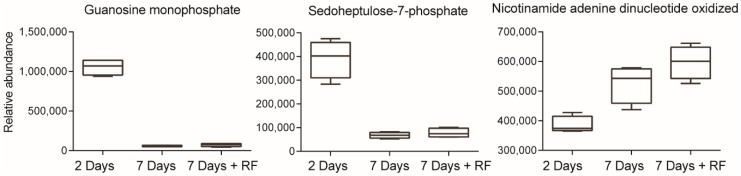
Multi-group analysis showing changes of the metabolome in MCF-7 cells treated with the vehicle (control). Cells were analyzed at two days, seven days and seven days with refeeding (*n* = 4/group). Box plots show relative abundances of guanosine monophosphate, sedoheptulose-7-phosphate and oxidized nicotinamide adenine dinucleotide oxidized changing over time.

To identify metabolic pathways modulated by the drugs, MCF-7 breast cancer cells (which are ER+) were dosed with either vehicle (control), palbociclib, fulvestrant, or a combination dose containing both drugs (Figure 1). Cells were harvested two and seven days post-dose, with another set harvested after seven days post-dose, with re-feeding on day four. This design allowed for the evaluation of short- and long-term responses to drug treatment. Untargeted mass spectrometry-based metabolomics was carried out on the cell lysates to analyze intracellular metabolites and the obtained data evaluated using the XCMS Online platform https://xcmsonline.scripps.edu [12,13,14]. The data revealed that metabolites in control samples (vehicle) changed in abundance over time due to expected cancer cell proliferation. This can be seen in Figure 2 which shows the relative abundances of guanosine monophosphate, sedoheptulose-7-phosphate, and oxidized nicotinamide adenine dinucleotide changing over time. Therefore, meta-analyses were used for further data evaluation, comparing altered metabolites from each drug treatment normalized to controls [15]. The putatively identified metabolites from those experiments were subsequently mapped onto metabolic pathways using mummichog analysis housed on XCMS Online [12,16]. Metabolite identities were confirmed by comparing MS/MS spectra with authentic reference standards, and quantified by targeted multiple reaction monitoring mass spectrometry, which provided additional validation.

**Figure 3:**
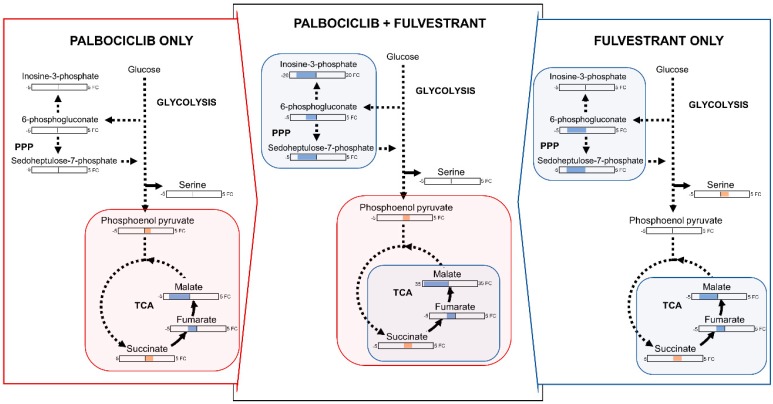
Overview of metabolite changes occurring in MCF-7 breast cancer cells two days post dosing with palbociclib, fulvestrant or in combination. Fold change values for significantly dysregulated metabolites (*p* < 0.05) are reported in orange (increase) and blue (decrease).

The results indicated that two days after a simultaneous dose of both drugs (palbociclib and fulvestrant) metabolites were dysregulated in multiple metabolic pathways in central carbon metabolism: The tricarboxylic acid (TCA) cycle, PPP, and purine synthesis. Figure 3 illustrates the changes to these pathways on single and combination dosing. In the TCA cycle, succinate was increased 1.9-fold, and fumarate and malate were decreased 2.0-fold and 34.0-fold respectively. This indicates a blockade of succinate metabolism possibly through inhibition of succinate dehydrogenase (SDH). Mutations to SDH have been seen in multiple cancers, with elevated succinate levels observed in cancer patients [17]. SDH requires FAD+ and NAD+ as co-factors [18], NAD+ was not decreased after two days of dosing, suggesting that other factors may be involved in SDH inhibition, such as tumor necrosis factor receptor-associated protein 1 (TRAP1). TRAP1 inhibits respiratory complex II downregulating SDH causing high concentrations of succinate [19]. This effect was also evident in single agent dosing of both drugs but to a lesser degree; malate was only decreased 4.0 and 3.5-fold when treated with palbociclib and fulvestrant alone respectively. Metabolites in both the oxidative and non-oxidative phases of the PPP were downregulated by combination dosing; sedoheptulose-7-phosphate (3.7-fold) and 6-phosphogluconate (2.5-fold). These metabolites were also down-regulated by fulvestrant dosing alone to a similar extent but were unaffected by palbociclib, therefore it appears that the combination drug dosing effects on the PPP are driven by fulvestrant alone. Furthermore, three additional metabolites with roles in central carbon metabolism, were altered after combination dosing but were not changed with any single agent dosing; N-acetylglucosamine phosphate (1.2-fold increase), inosine monophosphate (16-fold decrease), and fructose-1-phosphate (2.8-fold decrease) (Table S1). Given the changes observed to metabolites in PPP, purine synthesis, and TCA cycle intermediates after two days of dosing, it appears that these drugs act in synergy to target the same pathways that are important to cell growth and survival, as well as having separate actions on distinct pathways. This combinatorial effect enables a wider network of pathways to be modulated, thereby preventing the production of macromolecules and energy required for cancer cell growth and increasing senescence. A list of all significant metabolite changes after two days is illustrated in Table 1.

**Figure 4:**
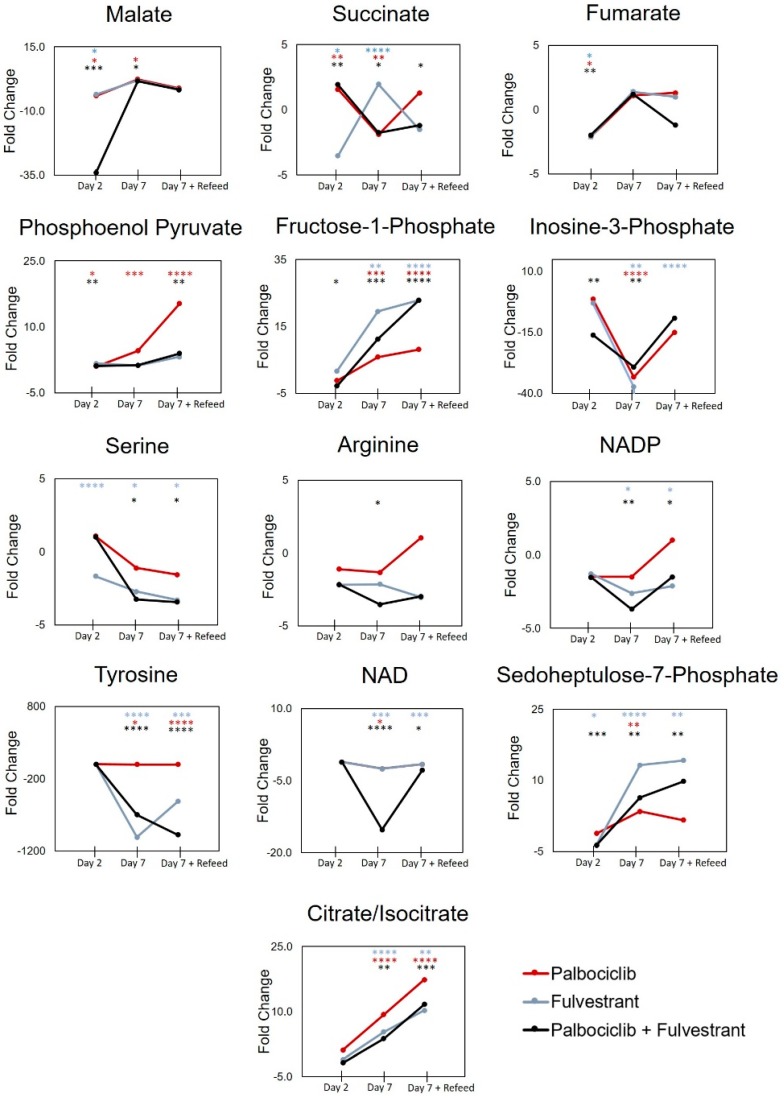
Fold change in metabolites in MCF-7 breast cancer cells over two, seven and seven days with refeeding, post palbociclib and fulvestrant, single and combination dosing compared to control. *p*-value statistical significance noted on graphs * < 0.05, ** < 0.01, *** < 0.001, **** < 0.0001.

At seven days post-dose of single and combination doses, changes to the metabolome were even more widespread than after two days. However, we first examined the effect of re-feeding the cells at day four to determine if the observed changes were due to nutrient depletion. It was seen that after refeeding, three metabolites were no longer dysregulated (malate, arginine, inosine monophosphate), therefore they were not altered because of drug efficacy and increased senescence (Figure 4, Table S1).

**Figure 5:**
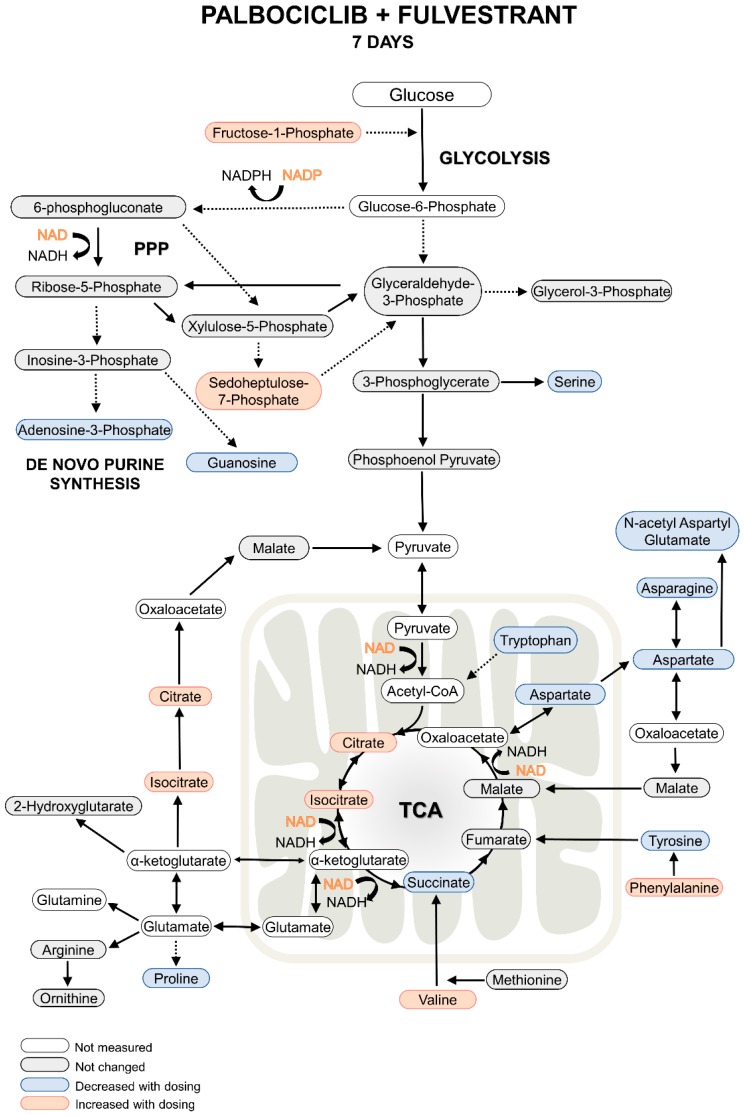
Overview of metabolite changes occurring in MCF-7 breast cancer cells. Seven days post palbociclib and fulvestrant, single and combination dosing.

There were however a number of metabolites that were changed after seven days of dosing and not affected by refeeding, as can be seen in Table S1. In contrast to the metabolic changes observed at day two post-dose, some of the metabolite concentrations were now changed in the opposite direction. For example, succinate decreased with both fulvestrant alone and combination dosing, however fumarate was not changed. This could be a result of decreased citrate/isocitrate utilization in the TCA cycle due to a now depleted NAD+; of note citrate/isocitrate were increased under all dosing conditions at seven days, which supports this hypothesis. Decreased citrate/citrate utilization in the TCA cycle would lead to lower levels of succinate. Succinate is known to upregulate TRAP1, therefore decreased succinate accumulation may impact SDH via decreased TRAP1 expression. Furthermore, deletion of TRAP1 has been shown to inhibit tumor growth in MCF-7 cells [20]. An additional change from two to seven days post-dose was seen in metabolites housed in the PPP; the intermediate sedoheptulose-7-phosphate was increased after fulvestrant and combination dosing. Fructose-1-phosphate was similarly changed, this metabolite can feed into the glycolytic pathway to produce glyceraldehyde and dihydroxyacetone phosphate, however additional changes to metabolites in the glycolysis pathway were not observed (or could not be measured) at seven days compared to controls. The depletion of serine (fulvestrant and combination) and tyrosine (palbociclib, fulvestrant, and combination), and an increase in phosphoenolpyruvate (palbociclib only) indicate that isoform M2 of pyruvate kinase (PKM2) regulation could be affected by the actions of palbociclib in single and combination doses [21]. At seven days post-dose, metabolite flow into the TCA cycle changed dramatically with an accompanying decrease in amino acids (aspartate, serine, proline, asparagine, tryptophan, tyrosine), NAD+ and NADP+ (Figure 5, Table 2). All these actions were driven by fulvestrant and could lead to increased oxidative stress in the cell thus causing cell death [22].

Taken together the results at seven days after combination dosing of palbociclib and fulvestrant show that the drugs act to inhibit purine, amino acid and important coenzyme synthesis. They also modulate glycolytic, TCA cycle and PPP metabolism possibly affecting the regulation of key enzymes such as PKM2 and SDH. An overview of metabolite changes after seven days with re-feeding is shown in Figure 5 and Table 2.

### 2.2. Transcriptomics Analysis

**Figure 6:**
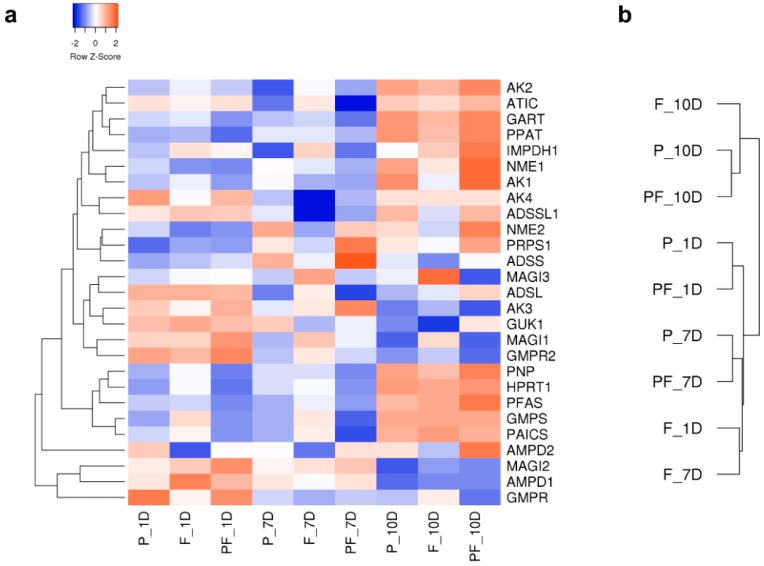
Differential regulation of selected genes after palbociclib (P), fulvestrant (F), and combination (PF) dosing, one day (1D), seven days (7D) and ten days (10D) post-dose: (**a**) heatmap of dysregulated genes (gene changes expressed as z-scores of the fold change), (**b**) cluster analysis of gene expression profiles.

In addition to comprehensive metabolomic analysis, RNA sequencing was performed to determine gene expression changes relating to single agent or combination drug dosing. Cells were collected at various time points (one day and seven days after dosing, with an additional time point at ten days with re-dosing at day seven). Metabolic genes appear highly over-represented among those whose expression were modulated by palbociclib as well as its combination with fulvestrant (Figure S1; Table S2). Gene set enrichment analysis [23,24] further identified significantly impacted metabolic pathways (Table S3) including metabolism of drug, nucleotides (both pyrimidine and purine; Figure S2), amino acids (histidine, phenylalanine and glycine/serine/threonine), fatty acids and retinol. The differential regulation of selected genes after palbociclib, fulvestrant, or combination treatment dosing compared to the control, can be seen on Figure 6 and Table S2.

To correlate our metabolite changes with gene expression changes, we used the recently developed systems biology, omic data integration tool on XCMS Online [12]. This tool identified four metabolic pathways which had both metabolite and gene involvement during combination dosing. These pathways are all associated with purine biosynthesis and degradation (Table 3). The two main genes identified in these metabolic pathways were phosphoribosylaminoimidazole carboxylase (PAICS) and purine nucleoside phosphorylase (PNP) which were both highly downregulated at all time points (Table S2). This confirms that combination dosing affects the biosynthesis of purine nucleotides. However, due to multiple single nucleotide polymorphisms that exist for both PAICS and PNP, interindividual variability in response to combination dosing may be seen in human populations. Other metabolic pathways altered by combination dosing were not identified at the gene level, suggesting the presence of a lag phase between gene-protein-metabolite expression or the insurgence of regulatory mechanisms directly operating at metabolite-protein level.

### 2.3. Comparison with Previous Results

Metabolomics analysis was previously conducted to characterize breast cancer cell line responses to clinical drugs such as the taxane-based chemotherapeutic paclitaxel [26]. Several metabolites such as aspartate, citrate, glutamate, pyroglutamate, myo-Inositol, UDP-glucuronate, and O-phosphocholine were increased in MCF-7 cells that were treated with paclitaxel. While branched chain amino acids such as leucine, isoleucine, and valine were reduced with paclitaxel treatment, metabolites in the glycolysis pathway, such as glucose and lactate were also decreased, which indicates a reduction in glycolysis with drug treatment. However, previous work focusing on single drug treatment and the response of combination therapy is sparse.

We previously identified enhanced disruption to nucleotide metabolism, amino acids, and PPP intermediates upon a two-day combination therapy with palbociclib and letrozole. Similar to the single treatment with palbociclib, minor changes were seen to cell metabolism; slight decreases in malate, no changes to amino acids and the majority of central carbon metabolites. Single treatments with letrozole and fulvestrant appeared to have different effects on MCF-7 cells, underscoring their different endocrine mechanisms; fulvestrant is a selective ER inhibitor, whereas letrozole inhibits estrogen biosynthesis. The metabolites with the strongest response to fulvestrant after two days of dosing were sedoheptulose-7-phosphate, serine, 6-phosphogluconate, fumarate, malate, succinate, cholesterol sulfate, and taurine. Whereas, only slight metabolic responses were seen with letrozole, with changes to 5-phosphogluconic acid, uridine, and metabolites in the glycolysis pathway.

Combination dosing with letrozole saw decreases in amino acids at two days, which were only apparent with fulvestrant at seven days post-dose. The major difference between the two treatments was the effect on nucleotide metabolism. In the case of fulvestrant combination dosing, we saw decreases in nucleotides, whereas with letrozole, increases were seen after two days of dosing, however it is not clear at this point whether a longer dosing time would result in similar effects. Thus, comparing these treatments show that combination therapies, which include a CDK 4/6 inhibitor and endocrine therapy, have a more profound effect on breast cancer cell metabolism.

## 3. Materials and Methods

### 3.1. Cell Culture

MCF-7 breast cancer cells (ATCC, Manassas, VA, USA) were cultured in RPMI 1640 medium (Gibco-Life Tech, Grand Island, NY, USA) supplemented with 10% fetal bovine serum (Sigma, St. Louis, MO, USA) and penicillin-streptomycin (Gibco-Life Tech, Grand Island, NY, USA) at 37 °C and 5% CO_2_. Cells were passaged routinely at a ratio of 1:3 or 1:4 every 3–4 days using trypsin/EDTA. For metabolomics experiments, cells were seeded into 150 mm cell culture dishes (Corning, NY, USA) and treated with either palbociclib (200 nM), fulvestrant (10 nM), a combination of those, or the vehicle as control. Four replicates were generated per experiment (approximately 3–5 million cells per replicate). Cells were harvested after two and seven days. Additionally, cells were taken after day seven with a refresh of the medium containing the respective drug(s) after day four. In total, there were *n* = 4/drug treatment groups at two, seven, and seven days with refeeding, resulting in a total number of 48 biological samples for metabolomics analysis.

### 3.2. Sample Preparation for Metabolomics Experiments

Prior to the sample harvest, cells were washed twice with phosphate buffer solution (Gibco-Life Tech, Grand Island, NY, USA) and 500 µL water was added to the culture dish. The bottom of the culture dish was flash-dipped into liquid nitrogen to quench metabolism immediately and cells were harvested using a cell scraper. Cell suspensions were transferred to 1.5 mL Eppendorf tubes and further processed with three freeze-thaw cycles (1 min freezing, 5 min thawing) on wet ice using liquid nitrogen and 10 min of sonication in an ice water bath. Samples were centrifuged for 10 min at 13,000 rpm and 4 °C. Supernatants were collected and protein concentration was quantified using a Pierce Micro BCA protein assay kit (Pierce, Rockford, IL, USA). Lysed samples were further extracted in acetonitrile/methanol/lysate (2:2:1 *v*/*v*/*v*). Tubes were then vortexed for 30 s in 1.5 mL Eppendorf tubes, sonicated for 10 min and stored at −20 °C for 1 h. Samples were subsequently centrifuged for 15 min at 13,000 rpm and 4 °C. Supernatants were transferred to 1.5 mL high recovery glass autosampler vials (Agilent Technologies, Santa Clara, CA, USA) and dried in a speedvac (Labconco, Kansas City, MO, USA). According to the protein concentration, the samples were resuspended in acetonitrile/water (50/50 *v*/*v*), where the lowest protein concentration was re-suspended in 100 µL, and all other samples were relatively adjusted thereafter. Samples were stored at −80 °C until analysis.

### 3.3. Untargeted Metabolomics Analysis

Analyses were performed using a high performance liquid chromatography (HPLC) system (1200 series, Agilent Technologies, Santa Clara, CA, USA) coupled with an electrospray ionization (ESI) source and a 6550 ion funnel quadrupole time-of-flight (Q-TOF) mass spectrometer (Agilent Technologies, Santa Clara, CA, USA). Samples were injected (8 µL) onto a Luna aminopropyl, 3 µm, 150 mm × 1.0 mm I.D. column (Phenomenex, Torrance, CA, USA) for HILIC analysis in ESI negative mode. HILIC was chosen to analyze predominantly central carbon metabolites as they are typically retained better by HILIC stationary phases upon comparison with reversed phase columns. Pooled samples were injected after three experimental samples, whereas one solvent blank was injected after every sample for QC. Mobile phase was A = 20 mM ammonium acetate and 40 mM ammonium hydroxide in 95% water, 5% acetonitrile and B = 95% acetonitrile, 5% water. The linear gradient elution from 100% B (0–5 min) to 100% A (50–55 min) was applied in HILIC at a flow rate of 50 µL/min. To ensure column re-equilibration and maintain reproducibility, a 10 min post-run was applied. ESI source conditions were set as follows: gas temperature 200 °C, drying gas 11 L/min, nebulizer 15 psi, fragmentor 365 V, sheath gas temperature 300 °C, sheath gas flow 9 L/min, nozzle voltage 500 V, and capillary voltage 2500 V. The instrument was set to acquire over a *m*/*z* range from 60–1000 with the MS acquisition rate of 1.67 spectra/s. For the acquisition of MS/MS spectra of selected precursors the default isolation width was set to narrow (1.3 Da), with a MS acquisition rate at 1.67 spectra/s and MS/MS acquisition at 1.67 spectra/s. The collision energy was set to 20 eV. Data was processed using XCMS Online [27] with a *p*-value of < 0.05 and *q*-value of < 0.1 set as statistical significance threshold cut-offs. Features were listed in a feature list table and as an interactive cloud plot, containing their integrated intensities (extracted ion chromatographic peak areas), observed fold changes across the sample groups, and statistical significance for each sample.

### 3.4. Targeted Metabolomics Analysis

For targeted analysis, a volume of 2 µL was injected onto a Luna aminopropyl, 3 µm, 150 mm × 2.0 mm I.D. column (Phenomenex, Torrance, CA, USA) using an Agilent Technologies series 1290 Infinity HPLC system with a gradient mobile phase of A = 20 mM ammonium acetate and 40 mM ammonium hydroxide in 95% water, 5% acetonitrile and B = 95% acetonitrile, 5% water. The linear gradient elution from 95% B (0–2 min) to 10% B for 13 min, then to 0% B in 2 min, held at 0% B for 3 min, and equilibrated back to 95% B over 4 min at a flow rate of 350 µL/min. The quantification of metabolites was performed by dynamic multiple reaction monitoring triple quadrupole mass spectrometry (Agilent Ion-Funnel 6490, Santa Clara, CA, USA). The ESI source conditions were as follows: Gas temperature 225 °C, gas flow 15 L/min, nebulizer 35 psi, sheath gas 400 °C, sheath gas flow 12 L/min, capillary voltage 2500 V (ESI negative) or 3500 V (ESI positive) and nozzle voltage 0 V. The cycle time was set to 500 ms. The collision energies, quantifier and qualifier ion transitions were optimized for each metabolite using MassHunter Optimizer software and are reported in the supplementary Table S1. To ensure accurate quantification, external calibration with standard compound mixtures was performed. Agilent QQQ Quantitative Analysis software was used to calculate the absolute concentrations of the metabolites in the samples.

### 3.5. Transcriptome Analysis

MCF-7 breast cancer cells were grown as above and collected at various time points (one day and seven days after dosing, with an additional time point at ten days with re-dosing at day seven), *n* = 3/group. Whole transcriptome RNA sequencing was performed by Biomiga (San Diego, CA, USA). Each treatment and time point combination was profiled in biological duplicates. The 50 bp paired end reads were mapped by bowtie2 [28] and quantified using RSEM package [29]. Differential expression statistics was determined with EdgeR algorithm [30] from expected counts. Gene set enrichment analysis [31] was performed using TPM values based on weighted signal-to-noise metric and false discovery rate (FDR) was assessed from 1000 permutations. Gene expression profiles cluster analysis was performed by average linkage with the Euclidean distance measurement method. Pathway gene signatures were prepared by mapping gene pathway list from KEGG database [23,24] to Entrez gene IDs at Pfizer.

### 3.6. Statistical Analysis

GraphPad Prism v 6.00 (GraphPad Software, Inc., San Diego, CA, USA) was used for statistical analysis. The quantitative triple quadrupole data was log transformed and expressed as mean ± standard error of the mean (S.E.M) after two-tailed *t*-tests were carried out. Comparisons with *p* < 0.05 were assigned to be statistically significant and noted on each graph.

## 4. Conclusions

To advance the understanding of single versus combination drug therapeutics on cancer cell metabolism, we used meta-analyses and a novel multi-omics technology to correlate metabolites and genes to decipher dysregulated metabolic pathways. This tool, which is freely available on XCMS Online, revealed several pathways significantly modified by palbociclib, fulvestrant, or a combination of the two. We observed that individually, palbociclib and fulvestrant caused disruption to shared metabolic pathways, however only fulvestrant acts on the PPP and has a more profound effect on amino acid biosynthesis. The combined effect of dosing with both drugs enables a comprehensive attenuation of metabolic pathways involved in co-enzyme production, energy metabolism, and macromolecule biosynthesis. This confirms that there is an enhanced effect of combined breast cancer therapy over single treatment, which was reported recently in a phase 3 study in vivo [7]. Interestingly, after seven days post-treatment, we observed the initiation of a salvage mechanism, whereby the glycolytic pathway is shunted towards the PPP, indicating that the cells may be attempting to synthesize serine and purines through an alternative mechanism. Thus, future studies should be designed to investigate the adaptive mechanisms of the cancer cells after removal of the drugs.

## Figures and Tables

**Table 1:** Panel of significantly altered metabolites 2 days after dosing with palbociclib (*n* = 4), fulvestrant (*n* = 4) or a combination dose of both palbociclib and fulvestrant (*n* = 4) when comparing dosed groups to control. Values are log transformed fold changes after quantification by multiple reaction monitoring, unpaired two-tailed *t*-test.

Metabolite Name	Palbociclib		Fulvestrant		Combination	
	Fold Change	*p*-Value	Fold Change	*p*-Value	Fold Change	*p*-Value
Succinate	1.6	0.0025	1.8	0.0441	1.9	0.0021
Malate	−4.0	0.0170	−3.5	0.0381	−34.0	0.0002
Fumarate	−2.1	0.0189	−2.1	0.0438	−2.0	0.0003
Phosphoenolpyruvate	1.1	0.0450	N.S	N.S	1.2	0.0014
6-phosphogluconate	N.S	N.S	−3.7	0.0255	−2.5	0.0496
Sedoheptulose-7-phosphate	N.S	N.S	−3.6	0.0335	−3.7	0.0003
Serine	N.S	N.S	1.7	<0.0001	N.S	N.S
Cholesterol sulfate	N.S	N.S	−5.2	0.0332	N.S	N.S
Taurine	N.S	N.S	−2.1	0.0380	N.S	N.S
Inosine monophosphate	N.S	N.S	N.S	N.S	−16.1	0.0052
Fructose-1-phosphate	N.S	N.S	N.S	N.S	−2.8	0.0172

**Table 2:** Panel of significantly altered metabolites 7 days after dosing with palbociclib (*n* = 4), fulvestrant (*n* = 4), or a combination dose of both palbociclib and fulvestrant (*n* = 4) when comparing dosed groups to control. Values are log transformed fold changes after quantification by multiple reaction monitoring, unpaired two-tailed *t*-test.

Metabolite Name	Palbociclib		Fulvestrant		Combination	
	Fold Change	*p*-Value	Fold Change	*p*-Value	Fold Change	*p*-Value
Sedoheptulose-7-phosphate	3.5	0.0076	13.2	<0.0001	6.4	0.0012
NADP	N.S	N.S	−2.6	0.0168	−3.7	0.0030
Citrate/Isocitrate	9.3	<0.0001	5.3	<0.0001	3.8	0.0017
Succinate	−1.9	0.0047	−1.7	<0.0001	−1.8	0.0356
Malate	2.4	0.0130	N.S	N.S	1.7	0.0323
Fructose-1-phosphate	5.8	0.0036	19.5	0.0002	11.2	0.0006
N-acetylaspartylglutamate	N.S	N.S	−3.4	0.0030	−2.6	0.0051
Aspartate	N.S	N.S	−2.6	0.0021	−2.3	0.0041
Serine	N.S	N.S	−2.7	0.0370	−3.2	0.0245
Proline	N.S	N.S	N.S	N.S	−2.5	0.0014
Asparagine	N.S	N.S	−5.5	0.0021	−8.4	0.0003
Tryptophan	N.S	N.S	N.S	N.S	−2.9	0.0134
Tyrosine	−10.0	0.0138	−1008.1	<0.0001	−696.5	<0.0001
Phenylalanine	N.S	N.S	4.2	0.0007	3.5	0.0008
Valine	1.8	0.0050	1.5	0.0003	1.7	<0.0001
Arginine	N.S	N.S	N.S	N.S	−3.5	0.0174
Guanosine	−1.3	0.0496	N.S	N.S	−4.5	<0.0001
Inosine monophosphate	−33.3	<0.0001	−37.3	0.0032	−29.2	0.0021
Adenosine monophosphate	−8.5	0.0008	−11.1	0.0017	−10.9	0.0019

**Table 3:** Active pathways mapped using untargeted metabolite and RNA-seq data at seven days post combination dose by XMCS Online. Pathway names provided by BioCyc Database Collection [25].

Pathway	Genes		Metabolites	
	Number/All	%Overlap	Number/All	%Overlap
Adenosine nucleotides degradation II	1/5	20	3/10	30.0
Guanosine nucleotides degradation III	1/3	33.3	2/9	22.2
Urate biosynthesis/inosine 5’-phosphate degradation	1/2	50.0	1/8	8.3
Inosine 5’-phosphate biosynthesis II	1/5	20.0	1/12	N/A

## Supplementary Materials

The following are available online at http://www.mdpi.com/2218-1989/9/1/7/s1, Figure S1: The enrichment of metabolic genes among those modulated by drug treatments, Figure S2: GSEA enrichment plot of pyrimidine metabolism and purine metabolism (FDR *q* = 4.92E^−5^ and 3.83E^−2^), 24 h combo treatment, Table S1: Metabolites observed after treatment of MCF-7 cells with either palbociclib (*n* = 4), fulvestrant (*n* = 4) or a combination dose of both palbociclib and fulvestrant (*n* = 4), Table S2: Differentially expressed metabolic genes from drug treatments (FDR ≤ 0.01 and FC ≥ 2), Table S3: Transcriptome-based gene set enrichment analysis result of the KEGG metabolism-related pathways, significant in at least one of the samples (False Discovery Rate < 0.05).
