# Supplementary material for: Palbociclib and Fulvestrant Act in Synergy to Modulate Central Carbon Metabolism in Breast Cancer Cells
Source: Metabolites. 2019 Jan 2;9(1):7. doi: 10.3390/metabo9010007 (PMC6359333; doi:10.3390/metabo9010007)
Supplement: Supplementary file 1 [file metabolites-09-00007-s001.zip › metabolites-393942-final-suppl/Supplemental Figures.pdf]

Supplemental Figures

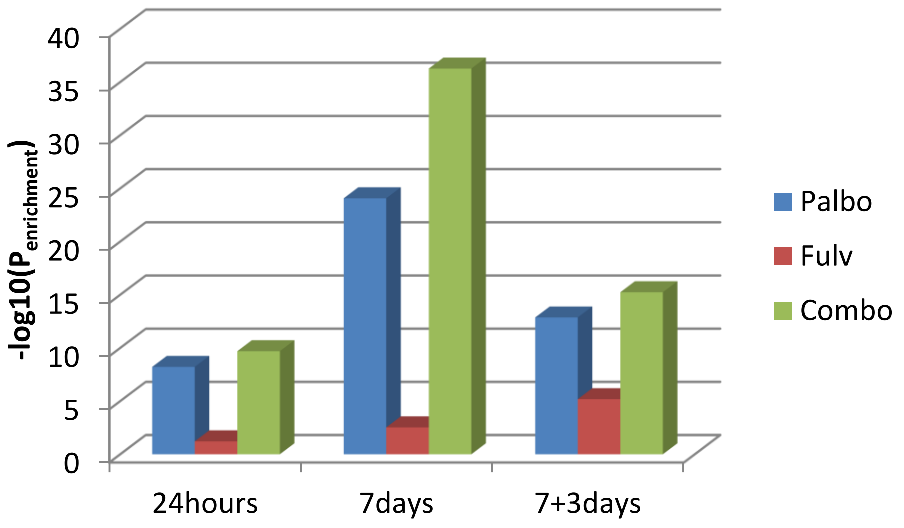

Figure S1. The enrichment of metabolic genes among those modulated by drug treatments. The statistical significance of over-representation was determined using cumulative hypergeometric distribution.

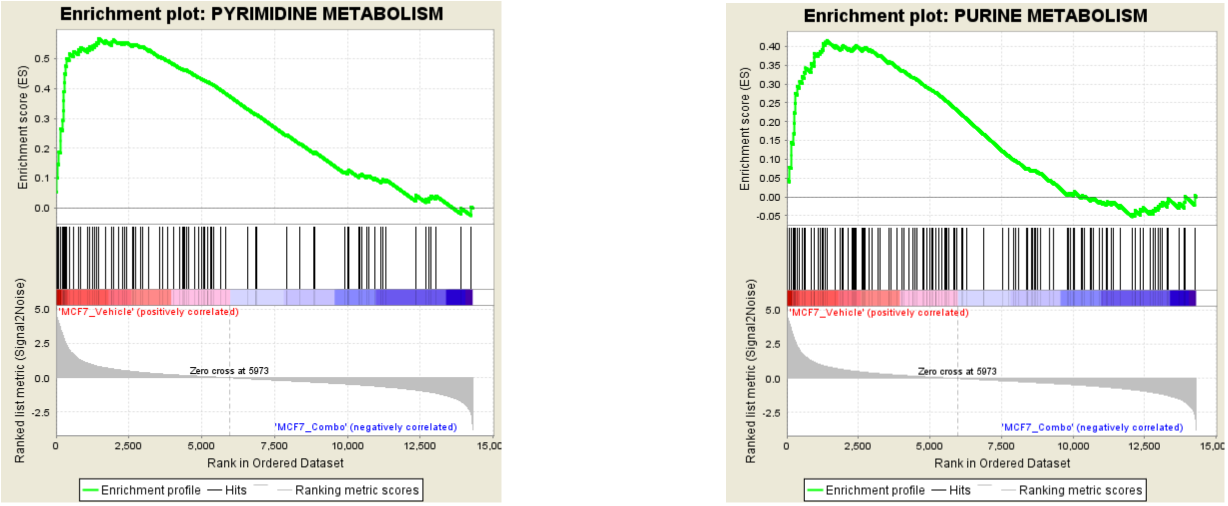

Figure S2. GSEA enrichment plot of pyrimidine metabolism and purine metabolism (FDR q = 4.92E^{-5} and 3.83E^{-2}), 24hr combo treatment.
